# Immediate modulation effects of Tongue Tri-needle on brain functional networks in infratentorial stroke patients with dysphagia: a randomized controlled trial

**DOI:** 10.3389/fneur.2025.1664668

**Published:** 2025-10-01

**Authors:** Fang Sun, Xiaoyan Huang, Jia Qiao, Lian Wang, Xue Cheng, Yan Chen

**Affiliations:** ^1^Department of Rehabilitation Medicine, People' Hospital of Yangjiang, Yangjiang, China; ^2^Department of Rehabilitation Medicine, The Third Affiliated Hospital of Sun Yat-sen University, Guangzhou, China; ^3^Department of Rehabilitation Medicine, Union Hospital, Tongji Medical College, Huazhong University of Science and Technology, Wuhan, China; ^4^Department of Rehabilitation Medicine, The First Affiliated Hospital, Sun Yat-sen University, Guangzhou, China; ^5^Department of Dermatology, People' Hospital of Yangjiang, Yangjiang, China

**Keywords:** stroke, dysphagia, Tongue Tri-needle, functional near-infrared spectroscopy, randomized controlled trial

## Abstract

**Background:**

Tongue Tri-needle has demonstrated clinical efficacy in post-stroke dysphagia, but its neuromodulatory mechanisms in infratentorial stroke patients remain unclear. This study aimed to investigate the characteristics of resting-state brain functional networks in infratentorial stroke patients with dysphagia and the dynamic modulation of brain functional networks induced by Tongue Tri-needle.

**Methods:**

Thirty eligible infratentorial stroke patients with dysphagia were randomly assigned to either the Tongue Tri-needle group or sham needle group. Functional near-infrared spectroscopy (fNIRS) was used to monitor brain activity across four experimental states. Graph theory analysis quantified changes in brain network functional connectivity (FC) and topological properties, complemented by clinical swallow function assessments.

**Results:**

Baseline analyses showed reduced functional connectivity between the fronto-temporo-parietal regions and the primary sensorimotor cortex, with the degree of reduction correlating with clinical impairment. Acupuncture specifically enhanced FC between frontal and temporal–parietal cortices, strengthened interhemispheric sensorimotor cortex connectivity, and significantly increased network centrality in the right dorsal lateral prefrontal cortex (DLPFC). During the electroacupuncture phase, frontotemporal-sensorimotor connections were further strengthened, whereas the post-needle resting state revealed adaptive reorganization of the network.

**Conclusion:**

Infratentorial stroke patients with dysphagia exhibit disrupted functional connectivity within the fronto-temporo-sensorimotor network, which is associated with clinical impairment. Tongue Tri-needle multi-stage, selective reconfiguration of brain functional networks, particularly by modulating the right DLPFC, a key hub, to promote functional integration of swallow-related neural networks. These findings provide a neuromodulatory mechanism supporting the use of Tongue Tri-needle for post-stroke dysphagia.

## Introduction

Dysphagia is a prevalent complication following acute stroke, significantly increasing the risks of aspiration pneumonia, malnutrition, mortality, and poor functional outcomes (1). Its severity is closely related to the lesion location. Since swallowing-related neurons are predominantly centered in the brainstem and there is a close association between the pontine region and corticobulbar projections, infratentorial stroke patients with dysphagia typically exhibit more severe dysphagia than those with supratentorial lesions (2, 3). Furthermore, brain remodeling patterns in subcortical versus cortical stroke patients differ markedly during rehabilitation. A functional MRI study that included both hemispheric and brainstem stroke patients found that in hemispheric stroke patients, changes in swallowing function were associated with functional connectivity changes in the ventral default mode network, whereas in brainstem stroke patients the changes were mainly related to the sensorimotor network of the left postcentral gyrus, suggesting fundamental differences in the underlying neural mechanisms (4, 5). This divergence may reflect widespread cortico-cortical and cortico-subcortical network disruption after stroke-related dysphagia, with neural plasticity-mediated reorganization playing a pivotal role in functional recovery (6).

In terms of treatment, tongue acupuncture, a specialized needling technique rooted in modern bioholographic theory, has demonstrated evidence-based efficacy in improving post-stroke dysphagia (7). Among its variants, the “Tongue Tri-needle,” a cornerstone of the “Jin’s Three-Needle” system developed by Prof. Jin Rui at Guangzhou University of Chinese Medicine, represents a prominent branch of tongue acupuncture widely used in Southern China for post-stroke dysphagia management. This technique involves two stimulation modalities: manual acupuncture and electroacupuncture. Electroacupuncture integrates pulsed electrical stimulation with traditional needling to enhance DeQi sensation. Anatomically, the needling targets lie within the submental triangle, encompassing the anterior belly of the digastric muscle, mylohyoid muscle, hypoglossal nerve, and cervical branch of the facial nerve (8). The recently proposed concept of the “neuro-acupuncture unit,” positing that acupoints constitute anatomical landmarks enriched with neural and neuroactive components (9). Neuroimaging studies have further shown that specific combinations of acupoints can activate distinct brain regions, suggesting that acupuncture-induced modulation of post-stroke neuroplasticity may constitute a key mechanism underlying its therapeutic effects (10, 11).

Functional near-infrared spectroscopy (fNIRS) has become a pivotal tool for investigating acupuncture mechanisms. Leveraging neurovascular coupling, fNIRS noninvasively measures concentration changes in oxygenated (HbO₂) and deoxygenated hemoglobin (HbR) to reflect cortical neural activity (12). Compared to functional magnetic resonance imaging (fMRI), fNIRS offers superior temporal resolution and enables real-time monitoring of brain functional dynamics during acupuncture in clinical settings, making it ideal for capturing needling-induced neurophysiological responses (13, 14).

In summary, due to the presence of critical anatomical structures for swallowing in subcortical regions, dysphagia is both highly prevalent and more severe following a subcortical stroke. Given that tongue acupuncture shows favorable clinical outcomes for post-stroke dysphagia, it is crucial to delve into the neural network alterations in subcortical stroke and the neural mechanisms underlying its functional improvements. This study utilizes multichannel fNIRS to investigate cortical functional activity patterns in infratentorial stroke patients with dysphagia, examining their relationship with swallowing impairment severity. Furthermore, we aim to elucidate the potential cortical response mechanisms induced by Tongue Tri-needle. The findings are expected to provide preliminary neurophysiological evidence supporting the clinical application of this therapy for post-stroke dysphagia management in infratentorial stroke patients.

## Patients and methods

### Procedure

**Figure 1:**
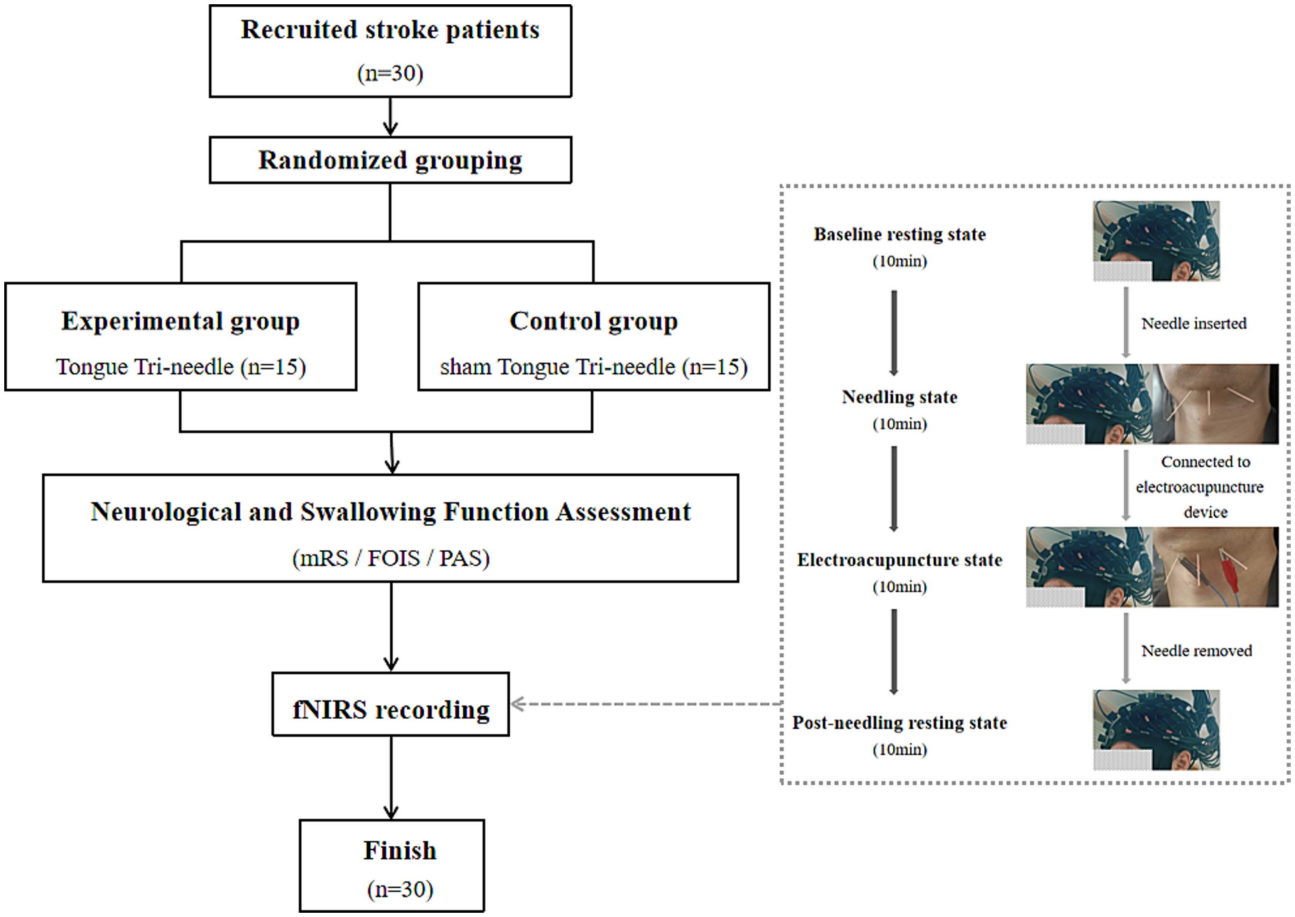
Experimental workflow.

All enrolled patients first completed standardized neurological and swallowing function assessments. Following function assessments, they underwent continuous fNIRS recordings captured cortical hemodynamic changes during baseline resting state, needling state, electroacupuncture state, and post-needling resting state, as shown in Figure 1. The study will be terminated immediately if any patient experiences discomfort during fNIRS recordings or acupuncture procedures.

Previous neuroimaging evidence has demonstrated that acupuncture elicits delayed neural effects, making conventional block-design paradigms unsuitable for capturing these temporally distributed responses (15). Therefore, we employed a non-repetitive event-related design to effectively dissociate the neural correlates of different acupuncture phases. Furthermore, based on established fNIRS methodology indicating that functional connectivity measurements require at least 7 min of continuous recording to achieve stability, we implemented a 10-min acquisition period for each experimental condition to ensure data reliability (16).

The experiment was conducted in a controlled environment with an ambient temperature maintained at 23 °C–26 °C to minimize potential errors in blood flow and heart rate measurements. Patients were instructed to abstain from alcohol and caffeine consumption for 24 h prior to the experiment to ensure consistency in physiological measurements. During the experimental procedures, all patients were instructed to remain awake and relaxed, and maintain minimal movement while seated.

### Patients

All infratentorial stroke patients with dysphagia were recruited from the Department of Rehabilitation Medicine of the Third Affiliated Hospital of Sun Yat-sen University between 01/12/2023 and 30/05/2024. Eligible participants met the following inclusion criteria: (1) MRI-confirmed ischemic or hemorrhagic stroke localized to the brainstem or cerebellum; (2) age between 18 and 80 years; (3) disease duration of 1–12 months; (4) dysphagia diagnosis confirmed by either Eating Assessment Tool-10 score ≥ 3 or videofluoroscopic swallowing study (VFSS); and (5) preserved cognitive function with the ability to maintain a seated position for 40 min (with or without assistance). Key exclusion criteria comprised: history of adverse reactions to acupuncture, impaired consciousness, tracheostomy status, unstable vital signs, structural abnormalities of the oropharynx or esophagus, severe comorbidities, concurrent neurological disorders (including encephalitis and multiple sclerosis), and pregnancy or lactation.

This study is a parallel-group randomized controlled trial assessing immediate post-intervention effects, employing a 1:1 allocation ratio and strictly adhering to CONSORT guidelines. Due to the absence of established sample size calculation standards for acupuncture neuroimaging studies, the sample size was determined based on previous neuroimaging investigations of acupuncture effects, which typically included 12–20 participants per treatment group (17, 18). Consequently, we enrolled 15 participants each in the experimental and control groups. The randomization procedure was conducted by an independent researcher using PASS software (15.0, NCSS, LLC), with a predetermined seed number to ensure reproducibility, and implemented through sequentially numbered, opaque sealed envelopes to maintain allocation concealment.

Given the inherent challenges in blinding practitioners and participants to acupuncture interventions, this study employed partial blinding whereby only outcome assessors and data analysts were masked to group allocation, with strict separation maintained between these personnel and the intervention team throughout the trial duration.

This study was conducted in accordance with the Declaration of Helsinki and the guidelines for human experimentation. It was approved by the Ethics Committee of the Third Affiliated Hospital, Sun Yat-sen University (NO. II2023-127-02). The study protocol was registered with the Chinese Clinical Trial Registry (No. ChiCTR2300073692). Written informed consent was obtained from all patients prior to their inclusion in the study.

### Neurological and swallowing function assessment

All swallowing function assessments were conducted by the same speech-language pathologist to ensure consistency. The modified Rankin Scale (mRS) was employed to evaluate post-stroke neurological functional status, while the Functional Oral Intake Scale (FOIS) was used to assess feeding capacity (19, 20). Patients who screened positive with EAT-10 scores ≥ 3 underwent subsequent videofluoroscopic swallowing study (VFSS) examination.

The standardized VFSS protocol was performed with participants in a comfortable seated position using a digital fluoroscopy system (Bluefin Imaging Athena Plus 7500, China) for lateral projection imaging. Following the modified Volume-Viscosity Swallow Test protocol (21), participants swallowed two boluses of nectar-thick liquid prepared with 60% w/v barium sulfate suspension. The contrast mixture was thoroughly homogenized and allowed to settle for 5 min prior to administration via syringe by either the patient or a family member. The examination was digitally recorded at 30 frames per second using a VFSS acquisition and analysis system (Longest Inc., Guangzhou, China), with aspiration severity evaluated according to the Penetration-Aspiration Scale (PAS), where scores ≥ 5 were considered indicative of aspiration (22).

### Tongue Tri-needle

**Figure 2:**
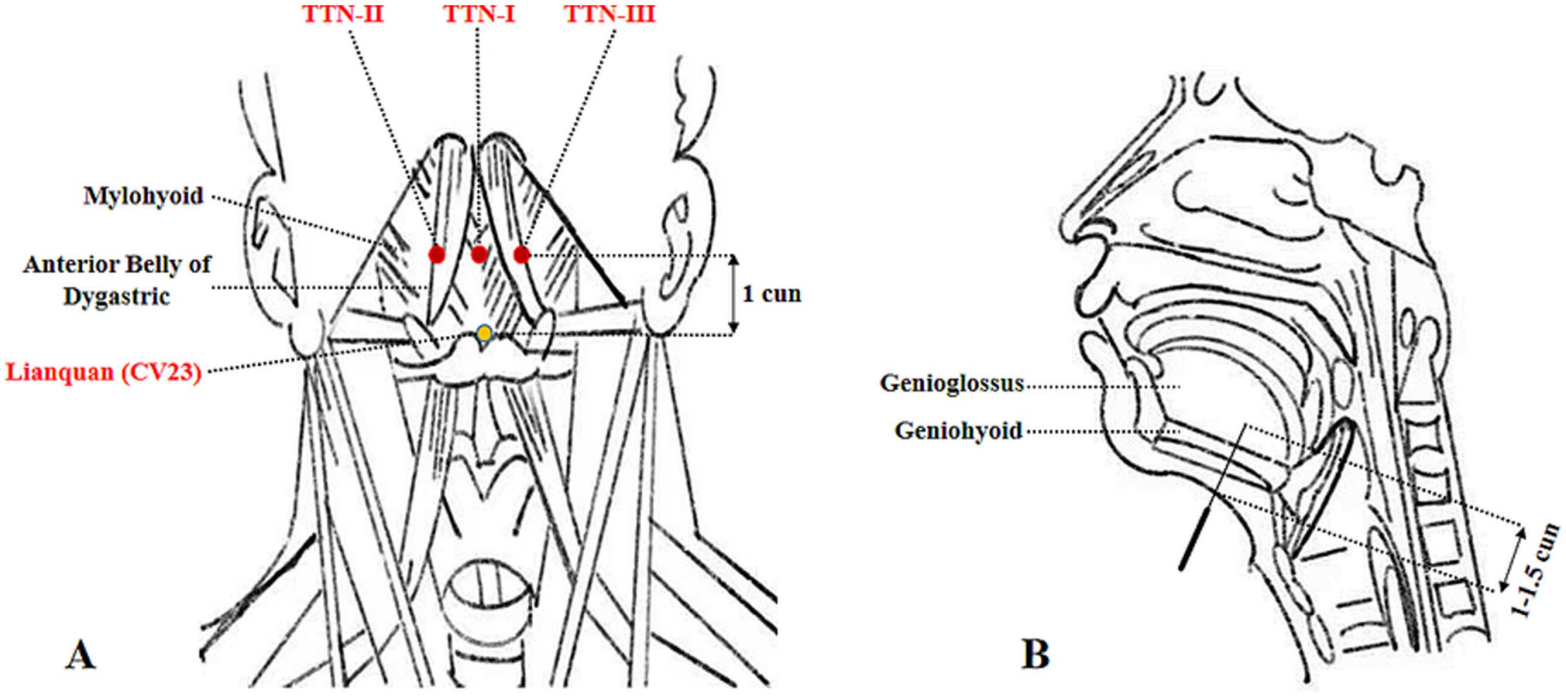
Anatomical schematic of Tongue Tri-needle acupoints and needle insertion parameters. **(A)** Anatomical localization of Tongue Tri-needle acupoints. **(B)** Needle insertion direction and depth.

Tongue Tri-needle was performed using sterile disposable stainless steel acupuncture needles (0.3 mm*40 mm). Needle placement followed the standard protocol described in reference (23). After routine skin disinfection, the first needle was inserted at Shanglianquan, located 1 cun above Lianquan on the anterior midline at the superior border of the hyoid bone, in the depression between the hyoid bone and mandibular border. The second and third needles were placed at bilateral Panglianquan, 0.8 cun lateral to Shanglianquan. All needles were directed toward the tongue root with an insertion depth of 1–1.5 cun (25–40 mm). The details of the acupuncture points are shown in Figure 2. For the electroacupuncture, the second and third needles were connected to an electroacupuncture device using dense-disperse waves (2/100 Hz) at an intensity tolerable to the patient.

The sham control group received placebo acupuncture using identical-looking needles with blunt tips that only touched the skin surface without penetration, secured at the same acupoints with adhesive collars. The electrodes were attached in the same manner as the treatment group but without electrical stimulation. To ensure consistency, all acupuncture procedures were performed by the same licensed acupuncturist with over 10 years of clinical experience.

As a well-established acupuncture technique, Tongue Tri-needle is minimally invasive. The primary adverse effects include localized bleeding at puncture sites and needle syncope, none of which pose life-threatening risks to patients.

### Functional near-infrared spectroscopy

**Figure 3:**
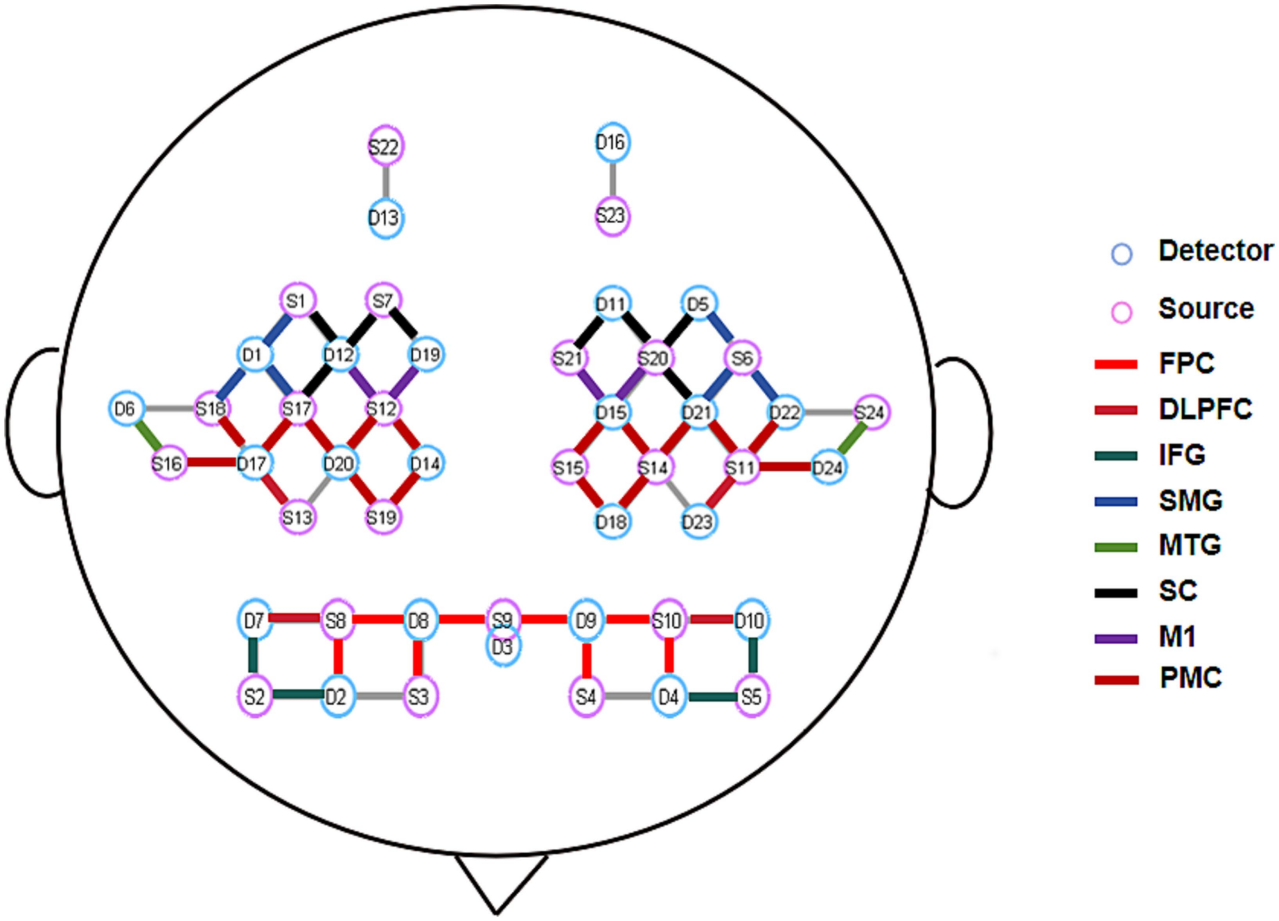
Schematic diagram of fNIRS optode arrangement and ROI distribution. FPC, Frontal Pole Cortex; DLPFC, Dorsolateral Prefrontal Cortex; IFG, Inferior Frontal Gyrus; SMG, Supramarginal Gyrus; MTG, Middle Temporal Gyrus; SC, Sensory Cortex; M1, Primary Motor Cortex; PMC, Premotor Cortex.

A continuous-wave fNIRS system (NirScan, Danyang Huichuang Medical Equipment Co., Ltd., China) was employed for hemodynamic data acquisition in this study. The system, operating at three wavelengths (740, 808, and 850 nm) with a sampling rate of 11 Hz, enabled real-time monitoring of oxygenated (HbO₂) and deoxygenated hemoglobin (HbR) concentration changes. A customized optode cap was positioned according to the international 10–20 EEG system, comprising 24 light sources and 24 detectors arranged with 3 cm inter-optode spacing to form 61 measurement channels (Figure 3). Channel configuration was standardized using a template-based approach, with three-dimensional digitization verifying optode placement and spatial registration performed according to the Montreal Neurological Institute coordinate system and Brodmann-Talairach atlas (24). Brodmann area labeling for each channel was determined by maximum probability mapping, with channel-wise data averaged within identical Brodmann regions.

The experimental protocol commenced with precise cap placement guided by four anatomical landmarks (bilateral preauricular points, nasion, and inion). Optode-scalp coupling was optimized through signal quality verification and gain adjustment prior to data collection. Continuous fNIRS recordings were obtained during acupuncture interventions to capture cortical activity dynamics across experimental conditions. Environmental light interference was minimized using a rigid optode holder and light-absorbing black cloth. Consistent with previous swallowing-related neuroimaging studies (25), our analysis excluded channels corresponding to primary auditory, visual, and frontal eye fields to reduce task-unrelated artifacts. Fifty-two channels covering eight bilateral regions of interest (ROIs) were retained for final analysis (Figure 2).

### fNIRS data processing

The raw fNIRS data were preprocessed using NirSpark (Danyang Huichuang Medical Equipment Co., Ltd., Danyang, China) following a standardized pipeline. Initial quality control involved inspection of all channels, with exclusion of those demonstrating poor signal quality. Subsequently, motion artifacts unrelated to the experiment were removed using the spline interpolation method. The optical density signals were bandpass-filtered at 0.01–0.2 Hz to eliminate uncorrelated noise components and baseline drifts, including contributions from heartbeat and respiration (26). Based on the modified Beer–Lambert law, the differential pathlength factor was set to [6 6], and the optical density data were converted into hemoglobin concentration data. Additionally, a short-separation channel with a 10 mm source-detector distance was employed to account for scalp blood flow changes.

### Functional connectivity

Functional connectivity (FC), which reflects the synchronization of hemodynamic activity between distinct brain regions, provides insights into potential neurofunctional coupling mechanisms through temporal correlations in blood oxygen level-dependent signals (27). In this study, FC analysis was employed to investigate the modulatory effects of Tongue Tri-needle on cortical functional networks. The NirSpark software was used to extract HbO₂ time-series data across four experimental conditions (resting state, needling state, electroacupuncture state, and post-needling resting state). Pearson correlation coefficients (*r*-values) were computed between 52 measurement channels and between eight bilateral ROIs. To meet the assumptions of parametric statistical analysis, correlation coefficients underwent Fisher’s z-transformation for improved normality. Subsequently, 52 × 52 (channel-level) and 16 × 16 (ROI-level) FC matrices were constructed.

FC strength under each experimental condition was quantified using matrix averaging, with *r*-values ranging from −1 to +1. Values approaching +1 indicate strong positive correlations, those nearing −1 represent strong negative correlations, and values around 0 suggest weak FC (28). This analytical approach enables comprehensive evaluation of acupuncture-induced modulations in functional network integration across different spatial scales.

### Graph theory-based analysis of global and nodal brain network topological properties

This study systematically investigated the regulatory effects of acupuncture intervention on brain functional network topology using graph theoretical approaches (29). The analysis was performed on the Gretna platform, where brain network models were constructed across a sparsity range of 0.1–0.9 with 0.05 increments. The selection of this sparsity range and increments was primarily based on methodologies established in previous studies investigating acupuncture’s modulation of brain functional connectivity (30, 31). Sparsity, defined as the ratio of actual connections to maximum possible connections, was selected based on previous evidence demonstrating its effectiveness in capturing dynamic topological changes across different states (16). Correlation matrices were converted to binary adjacency matrices for calculating network parameters at each sparsity level, followed by integration using the area under the curve (AUC) method to comprehensively characterize topological features of brain functional networks.

For global network properties, five key metrics were examined: (1) small-worldness (*σ*) evaluating simultaneous local specialization and global integration (σ > 1 indicating typical small-world organization); (2) clustering coefficient (Cp) measuring local connection density; (3) shortest path length (Lp) assessing information transfer efficiency; and (4) global (Eg) and (5) local efficiency (Eloc) quantifying information processing capacity at respective scales (32). These metrics collectively revealed fundamental organizational principles: higher Cp, γ, and Eloc values indicated stronger functional segregation, while lower Lp and *λ* with higher Eg reflected superior integration capacity.

At the nodal level, four topological parameters were analyzed: (1) degree centrality (number of direct connections); (2) betweenness centrality (hub role in information transfer, with higher values indicating crucial relay function); (3) clustering coefficient (local connection density); and (4) shortest path length (average connection efficiency to other nodes) (32). These nodal metrics provided critical insights into region-specific roles in network information processing.

### Statistical analysis

All statistical analyses were performed using SPSS 27.0 (IBM, United States) and MATLAB 2023 (MathWorks, United States). Normality of continuous variables was assessed using the Shapiro–Wilk test. Normally distributed data were presented as mean ± standard deviation (x̄ ± SD) and compared between groups using independent samples *t*-tests, while non-normally distributed data were expressed as median (interquartile range) [M (IQR)] and analyzed with Mann–Whitney U tests. Categorical variables were reported as counts, with between-group differences evaluated by chi-square or Fisher’s exact tests as appropriate.

FC matrix analyses were conducted in MATLAB, employing independent samples *t*-tests or Mann–Whitney U tests for group comparisons with a significance threshold of *p* < 0.01. For graph theory metrics, global network properties were compared at *p* < 0.05, while nodal measures underwent Bonferroni correction for 16 multiple comparisons (adjusted *p* < 0.003). Spearman’s rank correlation analyzed relationships between resting-state FC/graph theory metrics and swallowing function scores, with correlation coefficients (r) interpreted per established criteria: 0.9–1.0 (very high), 0.7–0.9 (high), 0.5–0.7 (moderate), and 0.3–0.5 (low) (33). All statistical figures were generated using GraphPad Prism 9.0 (GraphPad Software, United States).

## Results

Eligibility screening was conducted for 47 participants, resulting in 10 ineligible, 5 who declined participation, and 2 exclusions. A total of 30 participants were enrolled and successfully completed the study (Figure 1). No adverse reactions or dropouts occurred in either group of patients. Demographic and baseline clinical characteristics showed no statistically significant differences between the groups (*p* > 0.05) (Table 1).

**Table 1:** Demographic and functional characteristics of patients.

Variable	Experimental (*n* = 15)	Control (*n* = 15)	Tests statistic	*P*
Age (years), mean ± SD	55.00 ± 11.08	54.80 ± 10.36	0.051	0.960
Gender (male/female)	14/1	15/0	–	1.000
Disease duration (months)	3 (2, 4)	3 (1, 5)	−0.231	0.817
Stroke type (ischemic/hemorrhagic)	12/3	13/2	–	1.000
Lesion laterality (left/right/mixed)	6/4/5	6/3/6	0.234	0.890
Hypertension	10	10	–	1.000
Diabete	6	4	–	0.700
mRS	3 (2, 3)	2 (2, 4)	−0.180	0.857
PAS	4 (1, 8)	2 (1, 8)	−0.867	0.386
FOIS	2 (1, 3)	1 (1, 3)	−0.453	0.650

mRS, modified Rankin Scale; FOIS, Functional Oral Intake Scale; PAS, Penetration Aspiration Scale.

### Correlation analysis between resting-state FC and neurological functional scale scores

**Figure 4:**
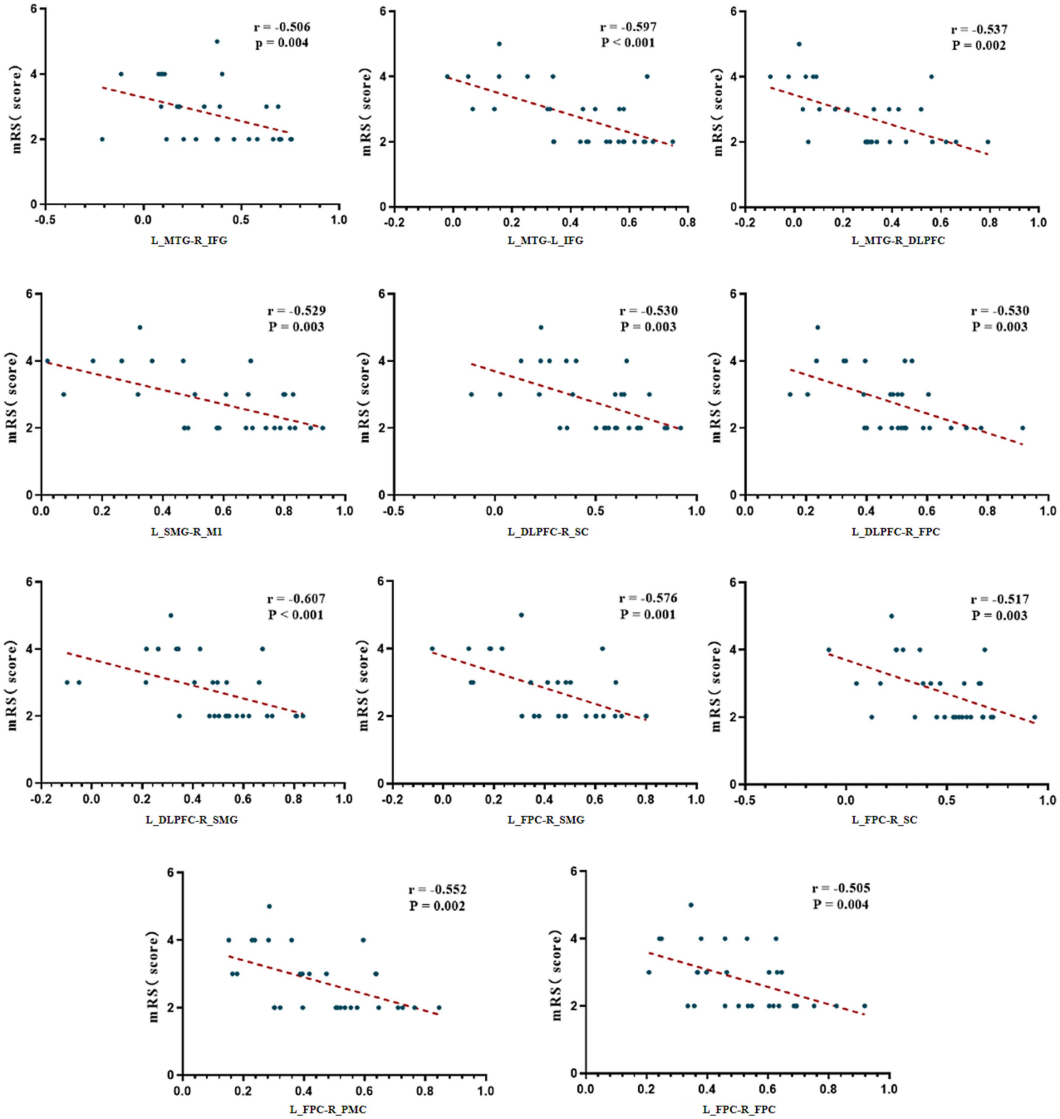
Correlation analysis between FC and mRS scores. mRS, modified Rankin Scale; L, Left; R, Right; FPC, Frontal Pole Cortex; DLPFC, Dorsolateral Prefrontal Cortex; IFG, Inferior Frontal Gyrus; SMG, Supramarginal Gyrus; MTG, Middle Temporal Gyrus; SC, Sensory Cortex; M1, Primary Motor Cortex; PMC, Premotor Cortex.

Spearman correlation analysis was used to assess the relationship between FC of various ROIs at resting state and baseline functional scale scores in all patients. The results showed that FC between brain regions had no significant correlations with baseline FOIS or PAS scores (*p* > 0.05). However, FC between multiple brain regions exhibited significant negative correlations with mRS scores (*p* < 0.05). Specifically, these included: left middle temporal gyrus (L_MTG)-(right inferior frontal gyrus) R_IFG, L_MTG-L_IFG, L_MTG-right dorsolateral prefrontal cortex (R_DLPFC), L_DLPFC-right sensory cortex (R_SC), L_DLPFC-right primary motor cortex (R_M1), L_DLPFC-right frontal pole cortex (R_FPC), L_DLPFC-right supramarginal gyrus (R_SMG), R_FPC-L_FPC, L_FPC-right premotor cortex (R_PMC), L_FPC-R_SMG, and L_FPC-R_SC (Figure 4). These findings suggest that weakened functional connectivity within the frontal-temporal–parietal network and the sensorimotor cortex is significantly associated with the severity of functional disability in patients with dysphagia following infratentorial stroke.

### Correlation analysis between resting-state brain network topological properties and neurological functional scale scores

**Figure 5:**
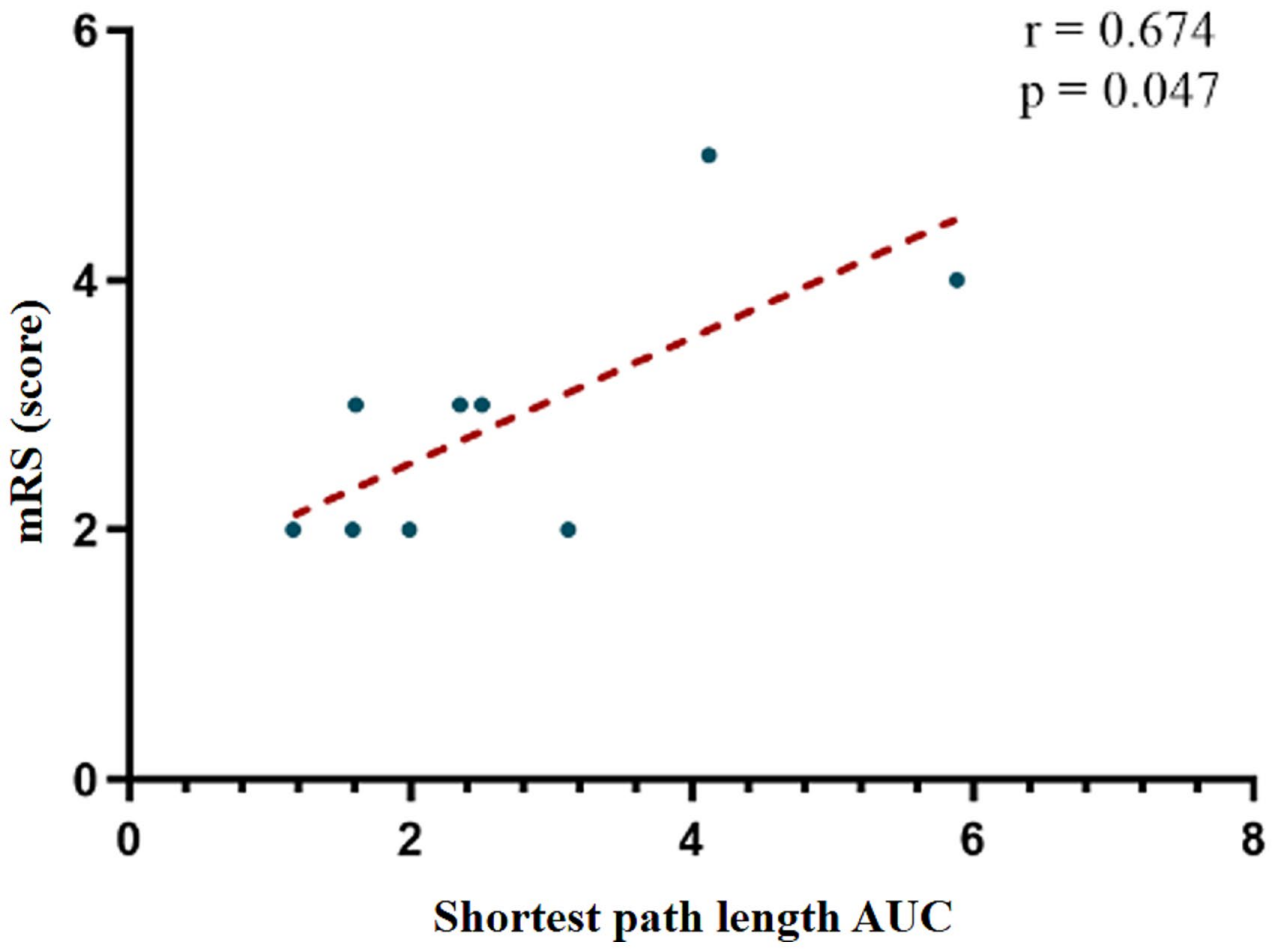
Correlation analysis between AUC values in R_IFG and mRS scores. mRS, modified Rankin Scale; AUC, Area Under the Curve.

Global network analysis revealed no significant correlations between small-world parameters and neurological functional scale scores (*p* > 0.05). At the nodal level, there was also no significant correlations between small-world parameters and FOIS or PAS scores (*p* > 0.05). However, a moderate positive correlation was identified between the AUC of the shortest path length in the R_IFG and mRS scores (*r* = 0.674, *p* = 0.047), suggesting that prolonged path length in this region may be associated with more severe functional impairment. This association remained statistically significant after correction for multiple comparisons (Figure 5).

### Differences in FC between the two groups

**Figure 6:**
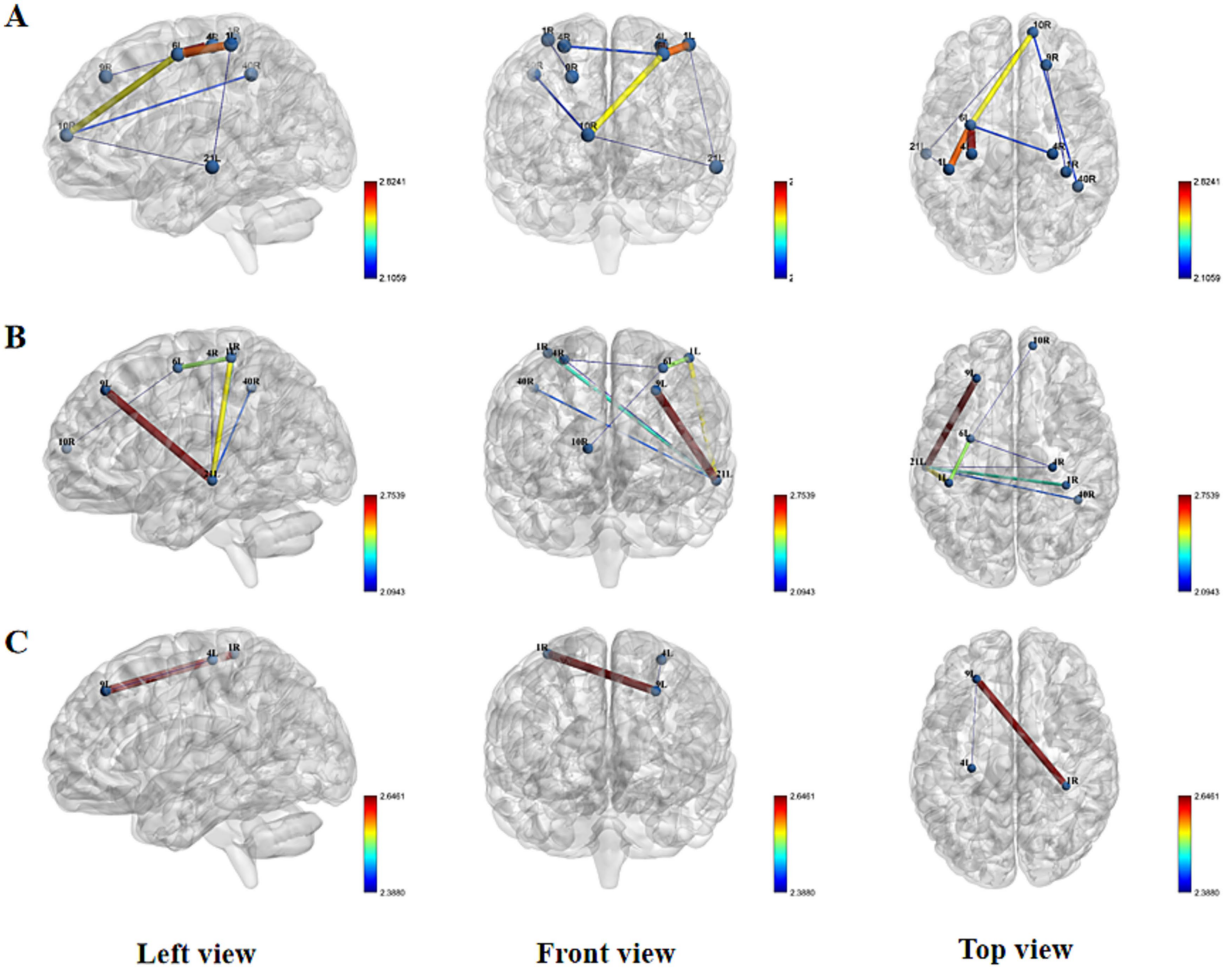
Spatial patterns of FC changes induced by Tongue Tri-needle. **(A)** Needling phase: intergroup differences in functional connectivity; **(B)** Electroacupuncture phase: intergroup differences in functional connectivity; **(C)** Post-intervention resting state: intergroup differences in functional connectivity.

At resting state, no statistically significant differences in FC were found between the two groups. However, distinct patterns emerged during different stimulation conditions. During the acupuncture state, the experimental group demonstrated significantly enhanced FC compared to the sham group in multiple connections, including R_DLPFC-R_IFG, R_IFG-L_MTG, L_PFC-L_MTG, L_MTG-L_SC, L_SC-L_PMC, L_PMC-L_M1, and L_PMC-R_M1 (Figure 6A). Comprehensive results are presented in the Supplementary Table S1.

This pattern of enhanced connectivity persisted during the electroacupuncture state, with the experimental group showing significantly increased FC in L_MTG-L_SC, L_MTG-L_DLPFC, L_PMC-L_SC, R_IFG-L_MTG, L_MTG-R_M1, R_M1-L_PMC, L_MTG-R_SMG, and R_PFC-L_PMC (Figure 6B). Comprehensive results are presented in the Supplementary Table S1.

Interestingly, in the post-acupuncture resting state, the real stimulation group exhibited significantly reduced FC in two specific connections: L_DLPFC-L_M1 and R_IFG-L_DLPFC compared to the sham group (Figure 6C; Supplementary Table S1). These findings suggest that acupuncture and electroacupuncture induce distinct patterns of functional connectivity changes during stimulation, with some effects persisting in a modified form during the post-stimulation resting state.

### Differences in brain network topological properties between the two groups

No significant between-group differences were observed in AUC values of small-world properties (*σ*, γ, λ) or other global topological metrics, including Cp, characteristic path Lp, Eg, and Eloc (*p* > 0.05).

However, during the needling phase, the verum acupuncture group demonstrated significantly elevated betweenness centrality AUC values in the R_DLPFC (6.683 ± 2.990) compared to the sham group (3.193 ± 2.415) (*T* = 3.518, *p* = 0.002). This difference remained statistically significant after Bonferroni correction (corrected *p* < 0.003), suggesting that the R_DLPFC assumed a more prominent hub-like role in the verum acupuncture group’s functional network architecture.

**Figure 7:**
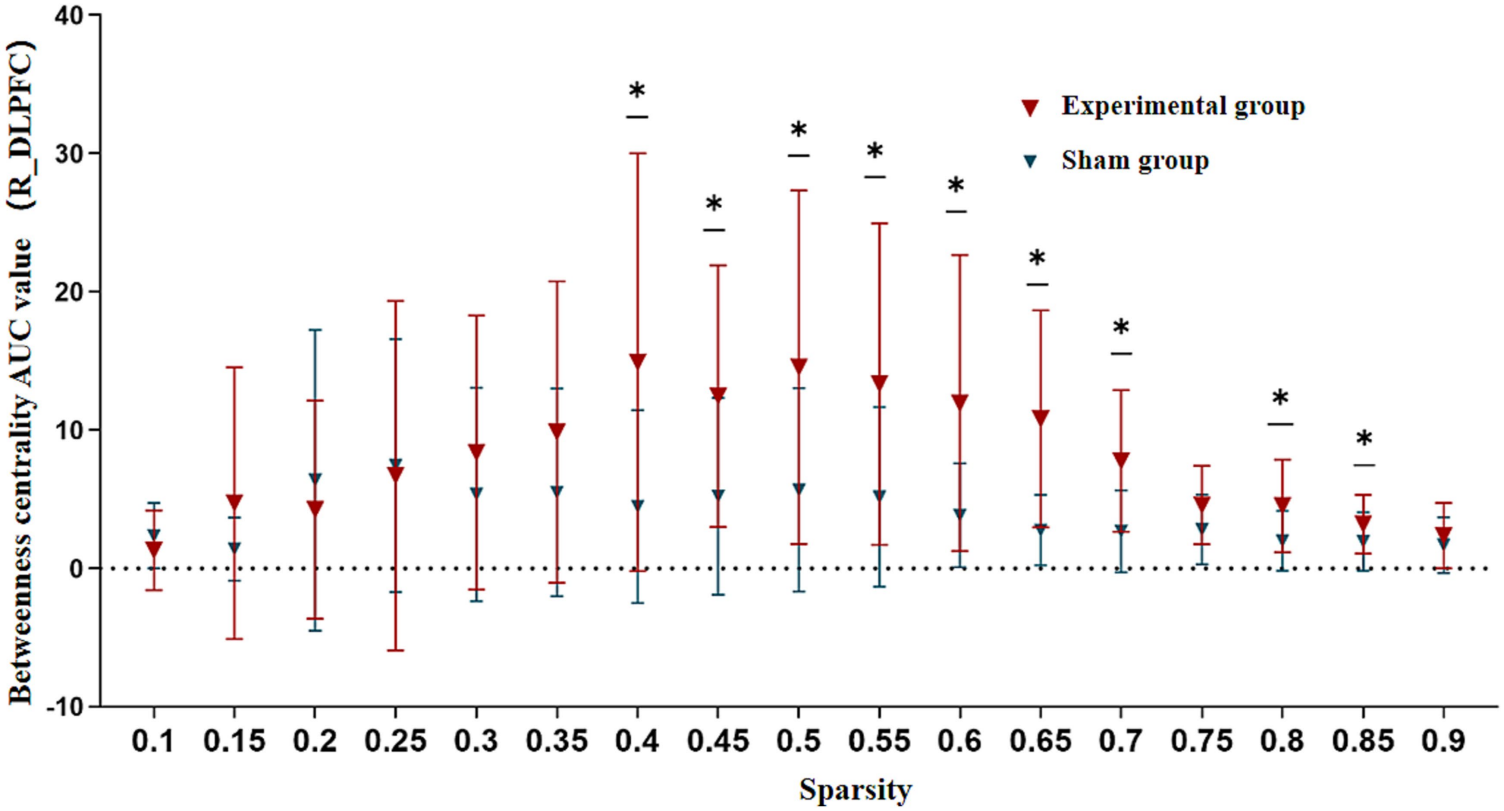
Betweenness centrality comparison in R_DLPFC between groups during needling phase. AUC, Area Under the Curve; R_DLPFC, Right Dorsolateral Prefrontal Cortex. Across 5–40% sparsity thresholds. *p < 0.05.

Additional non-parametric Mann–Whitney U tests revealed that this group difference in R_DLPFC betweenness centrality was particularly robust across a wide sparsity range (0.40–0.85), indicating consistent network reorganization effects regardless of connection density thresholds (Figure 7).

### Blinded assessment

After the trial, in the experimental group, 13 patients believed they had received acupuncture needle stimulation treatment, while 2 patients believed they had received sham stimulation treatment. In the control group, 11 patients believed they had received true acupuncture stimulation treatment, 2 patients were uncertain, and 2 patients believed they had received sham stimulation treatment.

## Discussion

The mechanisms underlying acupuncture therapy remain an important subject of investigation in neuroscience. Unlike conventional pharmacotherapy, acupuncture demonstrates unique “distant therapeutic effects” characterized by anatomical discontinuity between stimulation sites and target regions. Growing evidence suggests that acupuncture may promote functional recovery after stroke through modulation of cerebral neuroplasticity (11). Within this theoretical framework, we innovatively employed fNIRS to study swallowing dysfunction in patients with infratentorial stroke. This approach enabled systematic examination of cortical functional states in relation to swallowing performance while capturing, for the first time, specific cortical responses elicited by Tongue Tri-needle.

Our findings reveal several clinically relevant neuromodulatory effects. At baseline, FC strength between fronto-temporo-parietal and sensorimotor cortices showed a significant negative correlation with mRS, whereas the nodal shortest path length in the right inferior frontal gyrus exhibited a positive correlation with mRS. Following acupuncture intervention, we observed enhanced functional connectivity between right frontoparietal and bilateral SC, accompanied by increased network hub function in the right dorsolateral prefrontal cortex as reflected by elevated betweenness centrality AUC values. Of particular interest, electroacupuncture stimulation selectively strengthened synchronized activity between frontotemporal and sensorimotor regions. During the post-needling resting state, Tongue Tri-needle demonstrated significant normalization of excessive connectivity between frontoparietal and premotor cortices.

### Cortical functional network reorganization characteristics in post-infratentorial stroke dysphagia

The regulation of swallowing function involves a complex neural network system encompassing key brain regions including bilateral sensorimotor cortices, insula, cingulate gyrus, as well as premotor and supplementary motor areas (34). Post-stroke neuroplastic changes primarily manifest as functional reorganization resulting from an imbalance between energy metabolic demands and blood supply. Modern neuroimaging techniques provide novel approaches to observe the dynamic changes of this reorganization in terms of FC and brain network topology. Previous studies have demonstrated that the degree of neuroplasticity is closely associated with lesion severity and functional prognosis (35). Damage to critical nodes within brain networks significantly impairs information transfer efficiency, with this reduced connectivity showing a clear correlation with motor dysfunction in patients (36).

Significant FC impairments between fronto-temporo-parietal regions and sensorimotor cortices in infratentorial stroke patients with dysphagia were observed in our study. This finding aligns with previous research, confirming that remote cortical network connectivity abnormalities can occur following infratentorial stroke. Notably, while multiple fMRI studies have reported FC disturbances in sensorimotor-insula-putamen circuits and demonstrated positive correlations between precentral gyrus-medulla oblongata FC strength and swallowing function in post-stroke dysphagia patients, our study did not identify significant associations between cortical FC and dysphagia severity (3, 37). These discrepancies may stem from differences in study populations and the inherent limitations of fNIRS in detecting deep brain structures (38).

For brain network topology analysis, we employed graph theory, a powerful tool for complex network research. Healthy brains typically exhibit characteristic small-world network properties, combining high local specialization with global integration (39). In contrast, post-stroke brain networks often demonstrate abnormal reorganization, manifesting as decreased global network efficiency or a shift toward regular network configurations (40, 41). Our study revealed a positive correlation between the shortest path length of the R_IFG and mRS, suggesting this region’s pivotal role in network information transfer. This finding carries important clinical implications: as the cortical representation area for oral and pharyngeal sensation, the IFG’s extensive connections with sensorimotor cortices and brainstem may constitute a crucial neural substrate for swallowing function compensation following infratentorial stroke. The significant hemodynamic changes observed in bilateral IFG during active swallowing tasks further support its hub role in the swallowing network (42, 43). These findings provide novel insights into the neural mechanisms of post-infratentorial stroke dysphagia and offer theoretical foundations for clinical assessment and therapeutic strategy development.

### Neural mechanisms of Tongue Tri-needle in post-infratentorial stroke dysphagia

This study employed fNIRS to systematically investigate the modulatory effects of Tongue Tri-needle on cortical functional networks in infratentorial stroke patients with dysphagia. Our findings substantiate the holistic-local synergistic principle of acupuncture (10), demonstrating that specific acupoints exhibit corresponding relationships with distinct functional brain regions, while acupoints along the same meridian elicit similar cortical responses (44, 45). The results revealed that Tongue Tri-needle induces characteristic functional network reorganization: manual acupuncture predominantly enhanced FC between bilateral frontoparietal regions and sensorimotor cortices, whereas electroacupuncture preferentially strengthened FC between frontotemporal areas and sensorimotor cortices. These observations align with previous studies demonstrating acupuncture’s ability to specifically modulate FC in stroke patients, providing further evidence supporting the neural mechanism of acupuncture mediated through brain network regulation (11).

From a temporal dynamics perspective, our study revealed phase-specific neural activation patterns induced by Tongue Tri-needle. During acupuncture, we observed immediate enhancement of network connectivity, consistent with previously reported consistent neural activation patterns evoked by acupuncture (46). In the post-needling resting state, the attenuated FC between frontoparietal regions and PMC reflected functional reorganization of neural networks. This temporally specific neural response pattern provides empirical support for the “acupuncture multi-network modulation hypothesis,” suggesting that acupuncture may integrate effective connectivity across multiple functional networks through the hub role of the default mode network (47).

In graph theory analyses, we made significant findings regarding network topology. While no significant changes in global network properties were detected, nodal analysis demonstrated that Tongue Tri-needle intervention specifically increased the betweenness centrality (AUC values) of the R_DLPFC. This discovery carries three important implications. First, from a network topology perspective, high betweenness centrality indicates this region’s pivotal role in global information transfer. Second, from a functional neuroanatomical standpoint, the DLPFC not only participates in feeding behavior regulation, but its dense connections with the insula and PMC provide the structural basis for sensorimotor integration (46, 48). Third, considering functional lateralization, the R_DLPFC’s unique involvement in interoception processing and negative emotion regulation may offer a therapeutic target for the emotional components of dysphagia (49). Collectively, these findings suggest that the R_DLPFC serves as a central node in swallowing network reorganization induced by Tongue Tri-needle, mediating functional restoration through top-down regulatory mechanisms.

Based on the findings from this single-intervention study, we preliminarily propose a neural mechanism hypothesis for Tongue Tri-needle in post-infratentorial stroke dysphagia. Peripheral stimulation activates the R_IFG-L_MTG circuit, with the R_DLPFC as the key hub, enhancing cross-network integration among fronto-temporo-parietal regions and sensorimotor cortices. This hypothesis may not only explains the neural basis of immediate intervention effects but also provides a scientific framework for understanding long-term therapeutic benefits. However, its ability to account for clinical efficacy, particularly long-term therapeutic effects, remains to be directly validated through longitudinal studies that integrate behavioral assessments and clinical outcome measures. Notably, the stage-specific and disease-selective patterns of neural activation observed in this study suggest that investigating central mechanisms of acupuncture in specific patient populations may be of considerable significance. Future research should further explore how varying stimulation parameters influence brain network reorganization to inform optimization of treatment strategies.

For the blind assessment results, they are consistent with previous findings (50), indicating that in most cases, participants in the true acupuncture group correctly guessed that they had received the real treatment, while patients in the sham acupuncture group also tended to believe they had received the real treatment. For the control group, this represents a relatively ideal outcome for acupuncture blinding.

### Limitations

Our study has several obvious methodological limitations, and below we discuss the deficiencies identified in this study.

First, the fNIRS technology possesses inherent limitations. The most prominent constraint is its shallow penetration depth, which typically allows detection of only the most superficial neural activity in the cerebral cortex and makes it difficult to obtain hemodynamic information from deeper brain regions (such as the thalamus and basal ganglia). Consequently, our findings can only reflect superficial cortical activity, potentially overlooking critical changes in deeper structures that may play important roles in swallowing function.

Another limitation of this study is the relatively small sample size and the absence of *a priori* statistical power analysis. The limited number of participants may reduce statistical power, making it difficult to detect subtle or moderate true effects and increasing the risk of false negatives. Thus, nonsignificant findings should be interpreted with particular caution. This limitation underscores the need for future work to recruit larger samples, extend the intervention period, and integrate multimodal neuroimaging techniques to validate our results and elucidate the long-term neural modulation mechanisms of the tongue stimulation.

In the FC analysis, we conducted a large number of statistical comparisons, but the multiple comparison correction method used may not be sufficiently stringent, which to some extent increases the risk of false-positive results. The reported connectivity findings should be interpreted cautiously in this context and only after appropriate FDR correction and they should not be regarded as definitive conclusions. In future research, we will employ more rigorous correction methods to obtain more robust results.

Moreover, our study cohort exhibited a significant gender imbalance, which could introduce potential confounding factors since sex differences may be associated with brain function and treatment response. Therefore, the generalizability of our conclusions to different gender groups may be limited, and future studies should recruit more gender-balanced samples to confirm the current findings.

Finally, this study evaluated only the immediate effects of a single intervention; although no adverse events were observed, the safety of Tongue Tri-needle in stroke patients, especially those with intracerebral hemorrhage, warrants further investigation.

## Conclusion

This fNIRS study preliminarily investigated the characteristics of cortical functional network reorganization in infratentorial stroke patients with dysphagia and the neuromodulatory mechanisms of Tongue Tri-needle. The results revealed characteristic functional connectivity impairments between the fronto-temporo-parietal and sensorimotor networks, and the topology of the R_IFG was correlated with clinical assessment metrics. During the intervention, acupuncture-related manual stimulation and electroacupuncture appeared to exhibit distinct regulatory tendencies: manual stimulation predominantly enhanced connectivity between the bilateral frontoparietal-sensorimotor networks, whereas electroacupuncture tended to strengthen frontal-temporal-sensorimotor integration. Graph-theoretical analyses suggested a hub role for the right dorsolateral prefrontal cortex in the swallowing network, and changes in betweenness centrality during the intervention warrant attention. Post-intervention resting-state analysis showed a normalization tendency for abnormal hyperconnectivity between the frontal cortex and premotor areas.

This study provides preliminary evidence for the brain network mechanisms underlying post-stroke dysphagia and yields neural modulation patterns that may inform future hypotheses for precision acupuncture protocols. Given the small sample size and the single-intervention design, these findings require validation in large, longitudinal studies. Future studies should employ multimodal neuroimaging techniques combined with long-term intervention designs, along with the inclusion of functional change assessments, to further explore the potential relationship between neuroplastic changes induced by Tongue Tri-needle therapy and improvements in clinical symptoms.

## Data Availability

The raw data supporting the conclusions of this article will be made available by the authors, without undue reservation.
