# Supplementary material for: Immediate modulation effects of Tongue Tri-needle on brain functional networks in infratentorial stroke patients with dysphagia: a randomized controlled trial
Source: Front Neurol. 2025 Oct 1;16:1664668. doi: 10.3389/fneur.2025.1664668 (PMC12520968; doi:10.3389/fneur.2025.1664668)
Supplement: Supplementary file 1 [file Data_Sheet_1.pdf]

Table Comparison of FC Differences Between Experimental and Control Groups Under Different Conditions

State	Variable	Experimental (n = 15)	Control (n = 15)	Tests statistic	P
Needling state	LSC-LMTG	0.266	0.068	2.108	0.044
	LSC-LPMC	0.611	0.449	2.662	0.013
	LM1-LPMC	0.639	0.482	2.824	0.009
	RIFG-LMTG	0.278	0.089	2.096	0.045
	RM1-LPMC	0.598	0.458	2.236	0.034
	RIFG-RDLPFC	0.517	0.375	2.134	0.042
	LPFC-LMTG	0.326	0.166	2.106	0.044
	RPFC-LPMC	0.442	0.288	2.553	0.016
	RPFC-RSMG	0.529	0.394	2.225	0.034
Electro-acupuncture state	LMTG-LSC	0.334	0.061	2.512	0.018
	LMTG-LDLPFC	0.438	0.228	2.754	0.010
	LPMC-LSC	0.592	0.459	2.438	0.021
	RIFG-LMTG	0.306	0.097	2.366	0.025
	LMTG-RM1	0.310	0.112	2.110	0.044
	RM1-LPMC	0.586	0.465	2.115	0.043
	LMTG-RSMG	0.351	0.170	2.222	0.034
	RPFC-LPMC	0.537	0.341	2.090	0.045
Resting state	LDLPFC-LM1	0.341	0.529	-2.388	0.024
	RIFG-LDLPFC	0.322	0.506	-2.646	0.013
